# The Sunday Question

**Published:** 1892-06

**Authors:** 


### THE SUNDAY QUESTION.

Mr. B. F. Underwood, the well known literateur and lecturer, in a recent address on the Sunday Question, at Toronto, spoke as follows :

The voices of Christian preachers from Toronto and other Canadian communities are not infrequently heard at Sabbath conventions held in the United States against what they call Sabbath desecration, in favor of what they call keeping the Lord's Day. Their solicitude for the Puritanical observance of Sunday in my country is not surpassed by my interest in a rational settlement of the Sunday question in theirs. The principles of freedom, of justice, of common sense are not limited by the boundary lines which divide nations, and the discussion of questions of public interest involving the rights of man, raised above the passions and prejudices of mere party politics, and above the contentions of local factions, should be considered in the cool, unimpassioned light of the understanding from all the existing points of view. If the Toronto preacher believes that running cars and reading newspapers and visiting libraries and art galleries on Sunday are sins against God, sins that will result in the damnation of souls, that the only proper place to go on that day is the church, and that the only proper thing to do on that day is to read the Bible and hear him and the like of him tell them how good God is and how bad they are, and to repeat what Abraham, Isaac and Jacob, and Moses and David and Solomon did and said—I say if he believes in these things, it is his duty to preach them, and there is no place, if such teachings be true, more in need of such preaching than the City of Chicago in which I live. But since it is my conviction that such views in regard to Sunday observance are mere superstition, unworthy of rational minds, it is my duty to say this ; and I know of no large community in America, in which the agitation of this subject is more needed than Toronto—the stronghold of Protestant conservatism, which means intellectual rigidity, the same as does Roman Catholic conservatism. Intellectual rigidity is the sin against the Holy Ghost which is unforgivable, unredeemable, irreversible, because it entails loss to the living and to millions yet unborn.

